# Agreements between Approved Societies and Hospitals

**Published:** 1913-06-21

**Authors:** 


OUR
### NATIONAL INSURANCE ACT DEPARTMENT.

### LVII.—Agreements Between Approved Societies and Hospitals.

Ever since the Premier promised the introduction, early in the present session of Parliament, of an amending Bill to the National Insurance Act suggestions have been made in the press almost daily as to possible amendments. The suggestions have been of a very varied character, and all parties interested have taken their share in putting forward schemes which are calculated to meet in some way their particular requirements. The approved societies, for instance, have been pressing for simplification of the administrative machinery, such as the substitution of half-yearly for quarterly cards. The doctors, too, have put forward their requirements, including the demand for the stipulation of an income limit for insured persons. A number of those who are averse to the Act on economical and political grounds have even gone so far as to suggest the repeal of the compulsory provisions of the Act in their entirety.

Great care and discrimination will be required in the selection of the amendments that are practical and are real improvements. It is probably on this account, as well as on account of the congested state of Parliamentary business that the Government intend to send the Bill to a Standing Committee for detailed examination, rather than to have it discussed in Committee of the whole House. The course of procedure of sending the Bill upstairs, which was announced in the House by the Premier, is open to objection because the discussions there are not so freely reported as those in the House itself, and in consequence really vital matters may be allowed to escape attention, and there is no adequate opportunity to discuss points of detail on the third reading.

### Fuller Recognition of Hospitals Required.

One matter which we have consistently urged as of vital importance in the amendment of the Act ever since it became law is in regard to the position of the voluntary hospitals under the altered conditions brought about by the Act. Our suggestions have already been brought prominently to the notice of the Government, and we do not intend to allow matters to remain as they are even though it may be difficult, on account of the intended method of procedure, to attract public attention to the legitimate demand of the hospitals for fuller recognition of the services they render to insured persons.

Correspondence has recently taken place in the columns of the *Daily News*, from which it is clear that the hospitals are not receiving even the bare recognition which the Act at present claims to afford. This is the contention which we have consistently put forward, and which was fully substantiated in the very important account which we published last week from the information supplied by the authorities of the London Hospital.

### English Commissioners' Memorandum.

It is curious that the publication of this correspondence should be immediately followed by the issue of a most important memorandum on the subject by the English Commissioners. The concurrence of publication may be merely a coincidence, but it is, nevertheless, worthy of note. The memorandum deals with Section 12 of the Act, and the Commissioners announce that they have been frequently approached by approved societies with regard to the effect of that section which relates to the payment of sickness, disablement, and maternity benefits in cases where a member is an inmate of an institution of the type mentioned in the section. The actual wording of the section is important, and the text of sub-section (1) is as follows: "No payment shall be made on account of sickness, disablement, or maternity benefit to or in respect of any person during any period when the person to or in respect of whom the benefit is payable is an inmate of any workhouse, hospital, asylum, convalescent home, or infirmary, supported by any public authority or out of any public funds, or by a charity, or voluntary subscriptions, or of a sanatorium or similar institution approved under the Act."

In sub-section (2) certain exceptions are made with which we shall deal presently. It is to be noticed in the first place that the sub-section quoted precludes the payment of sickness benefit to the insured person when that person is an inmate of an institution supported by a charity or by voluntary contributions. This immediately raises the question as to what institutions come within this description, and the Commissioners, in the memorandum, say that the decision depends upon the circumstances of the case. "The fact that an individual inmate is wholly or partly maintaining himself in the institution would not be conclusive on this point. Where, however, the fees of patients or the subscriptions of the persons entitled to make use of the institution constitute its principal support, or where the institution is on a co-operative basis, the Commissioners are advised that the section does not apply."

In these circumstances it would appear that it would be advisable for an authoritative list to be compiled of the names of those institutions which are in fact included in the scope of this section.

### A Verbal Amendment Necessary.

The next matter to which it is necessary to call attention is the use of the word "during." It was, doubtless, intended that the payment of benefit should not be made in respect of the period during which the person is an inmate of the institution, but it is possible for the section to be read as meaning that the payment is not to be made during the period that the person is an inmate, and many societies, adopting this view, have been paying the benefit to the insured person on account of the period for which he has received institutional treatment, as soon as possible after he has left the institution. The Commissioners now say explicitly—though without the authority of an order—that a society must not make cash payments in the ordinary course to an inmate of any institution covered by the terms of the section, either before or after he leaves the institution in respect of the period during which he was an inmate. As this is the attitude of the Commissioners, and they apparently intend to act accordingly by disallowing cash payments made by approved societies in the circumstances, it is incumbent upon them to introduce a verbal amendment to the section in the forthcoming Bill to obviate any possible misconception in the matter.

### Relief of Dependants.

Section 12, sub-section (2) ,provides the means for the disposal of the sum which would have been payable except for the operation of sub-section (1). Some of these means are obligatory, while others are discretionary on the part of the society. Thus if the insured person has dependants it is obligatory on the society to apply the benefit in whole or in part for their relief or maintenance. Again, if the insured person is in receipt of sanatorium benefit in an institution and has no dependants the obligation is imposed on the society to which he belongs to pay the sickness or disablement benefit as the case may be to the Insurance Committee administering the sanatorium benefit.

In this connection it should be pointed out that under Section 79 the expression "dependants" in relation to any person, is defined as "such persons as the approved society or Insurance Committee shall ascertain to be wholly or in part dependant upon his earnings." This is a very wide definition, and it is perhaps only natural that the experience of approved societies leads them to take the view that insured persons without dependants are a negligible quantity. Against this, however, we have the statement of the London Hospital that records have been kept for two months, which show that the insured persons without dependants from one large society alone are sufficiently numerous to bring that hospital an annual revenue of £400 if the sickness benefit for these persons were paid by that society to the hospital.

### The Disposal of Benefit when there are no Dependants.

When the approved society determines that the member has no dependants the society may apply the sum in providing him with surgical appliances when necessary, or may otherwise expend it for his benefit. Commenting upon this provision the Commissioners say in their memorandum that "these latter words are intended to give the society a wide discretion in using the sum available for the best interests of the member in the particular circumstances of the case. Societies have, in certain circumstances, discretion to retain the whole or part of the benefit in their own funds, but they are bound to exercise this discretion reasonably, and the Commissioners do not suppose that societies will find many occasions on which the benefit could properly be withheld altogether." Thus, although, as pointed out above, the Commissioners have declined to sanction the payment of sickness benefit in cash to the insured person, they are prepared to agree to the application of the money for his benefit in almost any other form. In the case of large societies it is virtually an impossibility for the benefits to be dispensed except in cash, and as the majority of insured persons are members of large societies it would seem that the observations of the Commissioners just alluded to are mutually destructive.

### Model Form of Agreement between Societies and Hospitals.

As an alternative to the application of the benefit in this way the society may pay the whole or part of the money to the institution towards the member's maintenance, provided that an agreement has been made for the purpose with the institution. Up to the present the approved societies have declined to enter into such contracts, although it is understood that they have been approached in the matter by the hospital authorities. The reason given by the approved societies is that as the section does not compel them to make payments to the hospitals in the circumstances, the societies which did so would be at a disadvantage as compared with the society which did not in the competition for members. This is, of course, a debatable point, but the fact is that the societies have not acted together and the hospitals have suffered in consequence.

It would now appear that the Commissioners are open to a modification of the generally accepted meaning of the sub-section in regard to the making of agreements, for they have supplied in the memorandum a simple form of agreement which may be used in the circumstances. Instead of it being a formal agreement between the society and the hospital as to the terms upon which the members of the society would be treated, the agreement is drafted in the form applicable to an individual member. This is a very important distinction, and opens up vast possibilities, for if the hospitals agree among themselves to approach the approved societies in regard to every case admitted to the wards when the person has no dependants, it is clear that in a number of cases, and particularly so far as the large societies are concerned, they may be only too glad to enter into the agreements in view of the remarks of the Commissioners as to the disposal of the benefit to the member in cash.

In any event the societies and the hospitals have now been favoured with the considered opinion of the Commissioners that Section 12 was intended to operate for the benefit of the funds of the hospitals, and that it is not to be allowed to become a dead letter.

